# Chemical Changes in Nonthermal Plasma-Treated N-Acetylcysteine (NAC) Solution and Their Contribution to Bacterial Inactivation

**DOI:** 10.1038/srep20365

**Published:** 2016-02-02

**Authors:** Utku K. Ercan, Josh Smith, Hai-Feng Ji, Ari D. Brooks, Suresh G. Joshi

**Affiliations:** 1 Department of Surgery, Center for Surgical Infection and Biofilm, Drexel University, Philadelphia, PA 19102 USA; 2 Department of Chemistry, Center for Surgical Infection and Biofilm, Drexel University, Philadelphia, PA 19102 USA; 3 Department of Microbiology and Immunology, Center for Surgical Infection and Biofilm, Drexel University, Philadelphia, PA 19102 USA

## Abstract

In continuation of our previous reports on the broad-spectrum antimicrobial activity of atmospheric non-thermal dielectric barrier discharge (DBD) plasma treated N-Acetylcysteine (NAC) solution against planktonic and biofilm forms of different multidrug resistant microorganisms, we present here the chemical changes that mediate inactivation of *Escherichia coli*. In this study, the mechanism and products of the chemical reactions in plasma-treated NAC solution are shown. UV-visible spectrometry, FT-IR, NMR, and colorimetric assays were utilized for chemical characterization of plasma treated NAC solution. The characterization results were correlated with the antimicrobial assays using determined chemical species in solution in order to confirm the major species that are responsible for antimicrobial inactivation. Our results have revealed that plasma treatment of NAC solution creates predominantly reactive nitrogen species versus reactive oxygen species, and the generated peroxynitrite is responsible for significant bacterial inactivation.

The antimicrobial effect of direct non-thermal plasma treatment is has been widely investigated and is becoming a well- known phenomenon^1^,^2^,^3^,^4^,^5^,^6^. There are different factors and mechanisms which influence the antimicrobial effect of non-thermal plasmas. The most common routes of microbial inactivation are reported as: UV photons generated during plasma discharge, plasma generated reactive oxygen species^2^ and reactive nitrogen species^1^ in the gas phase and diffusion of these species through the bacterial cell wall and membrane, physical effect of electron discharge, electrical field, UV and reactive species that cause damages to the cell surface, and localized heating effects to cell surface. All these routes are capable of inactivating microorganisms synergistically^5^,^7^,^8^. Recently, plasma treated liquids have shown an increasing interest due to their stable antimicrobial activity even up to two years^9^. Different groups have reported antimicrobial properties of liquids including water, 0.9% saline solution and phosphate-buffered saline (PBS) that are treated with different non-thermal plasma sources. The acidification of liquids following plasma treatment is one of the most commonly reported chemical modifications^9^,^10^,^11^,^12^,^13^,^14^,^15^,^16^,^17^,^18^,^19^,^20^. In previous publication, our group defined fluid-mediated plasma treatment, where a particular fluid is treated with a plasma discharge and then exposed to microorganisms in order to achieve microbial inactivation^9^. In fluid mediated plasma treatment, bacteria don’t come in contact with UV and electron discharge. Therefore the interaction of plasma generated electrons and UV radiation with the fluid being treated, and the subsequent diffusion of plasma generated ROS and RNS into treated liquid are thought to be the main cause for the antimicrobial effect^9^. The decreased pH of plasma treated liquid is attributed to generation of HNO_2_, HNO_3_ and, H_3_O^+^ 9,12,16. The acidification of plasma treated liquid is critical for a microbiocidal effect; however, it is reported that acidic pH is not the main source of antimicrobial effect^10^,^20^,^21^. Ikawa *et al*. have reported that acidic pH is essential for the antimicrobial effect of plasma treated liquids. They have demonstrated that when the pH is below 4.7, the antimicrobial effect of plasma-treated liquid is significantly higher compared to liquid with a pH greater than 4.7. They claimed that pH 4.7 is the critical value for microbial inactivation and it is almost universal for different types of bacteria including Gram-positive, Gram-negative, aerobic and anaerobic bacteria^14^. In addition to decreased pH, also nitrate (NO_3_^−^), nitrite (NO_2_^−^) and hydrogen peroxide (H_2_O_2_) have been detected in plasma treated liquids^16^,^22^,^23^. Plasma also generates other ROS and RNS such as OH radical, superoxide, and nitric oxide^3^,^7^,^11^. A direct interaction of these species in liquid with the electron discharge, and the plasma generated ROS and RNS in the gas phase and their diffusion into liquid may be responsible for the presence of NO_3_^−^, NO_2_^−^ and H_2_O_2_ in plasma treated liquids. Reactions of ROS and RNS lead to the formation of other species that might contribute to antimicrobial effect. It is reported that plasma generates NO and superoxide (O_2_^−^), and their interaction yields to peroxynitrite (ONOO^−^) formation^3^,^7^,^11^,^24^,^25^,^26^. Another possible route for peroxynitrite formation in plasma treated liquids is the formation of nitrosooxidanium (H_2_NO_2_^+^) via the reaction of H^+^ cation with NO_2_^−^ anion in a highly acidic environment. Nitrosooxidanium (H_2_NO_2_^+^) is broken down into nitrosonium (NO^+^) a cation that is highly reactive and can attack biomolecules in the cell, and form peroxynitrous acid (ONOOH) in the presence of hydrogen peroxide. Finally peroxynitrous acid is dissociated to peroxynitrite in aqueous medium^24^,^25^. Lukes *et al*. have shown the formation of NO_2_, NO., and OH. radicals and NO^+^ ions by plasma operated in ambient air at the gas-liquid interface, and peroxynitrite generated in plasma treated water which played a significant role in the antimicrobial activity of plasma treated water^26^. Peroxynitrite is highly reactive and can easily diffuse through the cell membrane due to its high permeability. It attacks various biomolecules in the cell and causes protein and lipid nitrosylation and also intracellular oxidation. Intracellular damage induced by peroxynitrite usually can’t be restored by cellular repair mechanisms and cells die^27^,^28^,^29^. Acidified nitrite and nitrate have been known for decades for their antimicrobial effect both *in vitro* and as a part of natural protection mechanism of the body^30^,^31^,^32^. Salivary nitrite comes in contact with the acidic content of the stomach when swallowed and acts as a natural host defense mechanism through the formation of biocidal species^33^. The antimicrobial effect of acidified nitrite is closely related to RNS involving mechanisms. For example, NO release from skin has been reported. Also natural flora of the skin reduces nitrate to nitrite. In the acidic milieu of the skin RNS, including nitrous acid (HNO_2_), dinitrogen trioxide (N_2_O_3_) and peroxynitrite (ONOO^−^) are produced via NO and nitrite and nitrate that act as non-specific protection against pathogens on the skin^34^. Various groups have demonstrated that acidified nitrite has antimicrobial effect on various skin and oral pathogens^33^,^34^,^35^. The composition of the liquid being treated should be considered as an important factor in order to correlate the mechanism of antimicrobial effect. As opposed to plasma-treated water and PBS, Oehmigen *et al*. have reported the antimicrobial effect of plasma-treated 0.85% saline solution (NaCl). However they concluded that effect of chlorine species that arises from Cl^−^ ion could be neglected^15^.

Previously our group has reported that N-Acetylcysteine (NAC) solution gains antimicrobial effect when treated with non-thermal, atmospheric dielectric barrier discharge (DBD) plasma (operated in ambient air, without using technical gases). NAC solution was capable of inactivating a broad range of multi drug resistant (MDR) bacteria and fungi in their planktonic and biofilm forms. It was also observed that although the pH of the solution dropped to the acidic side (pH 2.35), it was not the major reason for microbial inactivation^9^. During the same preliminary study, generation of hydrogen peroxide was detected (0.42 mM, 1.67 mM and 0.93 mM respectively, after 1-minute, 2-minutes and 3-minutes of non-thermal atmospheric DBD plasma treatment). The interesting observation was that the hydrogen peroxide concentration reached to saturation after 2 minutes of plasma treatment and then dropped after 3 minutes of plasma treatment^9^.

We hypothesized that plasma treatments of NAC solution generate RNS and ROS and their interactions further give rise reactive product which in the presence of plasma-induced acidic pH gets stabilized and exhibit strong antimicrobial properties. Therefore we set out to detect the major reactive species and to characterize their intermediate chemical species and correlate these interactive species with antimicrobial efficacy. In the present study the techniques such as nitrite and nitrate detection, UV-vis spectrum analysis, FT-IR analysis, NMR analysis, were performed in order to evaluate chemical modifications in NAC solution following non-thermal atmospheric DBD plasma treatment. Also, NAC solution was separated to its constituents by evaporating liquid phase. Chemical characterization was also performed for remaining NAC powder and evaporated liquid phase following separation of plasma treated NAC solution in order to understand chemical modifications in the constituents of plasma treated NAC solution. Thus, through a combination of physico-chemical analysis we predicted that during plasma treatment of NAC solution, ROS and RNS are formed in both gas phase and liquid phase. Low pH is the common result of the liquid-mediated plasma treatment. Even though high acidity doesn’t contribute to the antimicrobial effect, it is an essential component for the microbial inactivation. The antimicrobial effect of this solution originates from diffused ROS and RNS in liquid as opposed to direct plasma treatment, where physical impacts such as UV, electrical field, and electron bombardment are major contributors to the biocidal effect.

## Materials and Methods

### Nonthermal Plasma settings and NAC Treatments

Plasma treatment of NAC solution was carried out as previously explained^9^. In brief, a custom-made glass liquid container was built which can hold 1 mL of liquid and can maintain 1 mm of liquid column. Plasma treatment was performed for 1, 2 and 3 minutes with a DBD electrode (38 mm × 64 mm in size) that was placed above fluid holder with 2 mm discharge gap. Plasma treatment parameters were fixed as 31.4 kV and 15 kHz, which yield 0.29 W/cm^2^ power distributions.

NAC solution was prepared from 100 mM stock solution. Stock solution was prepared by dissolving NAC powder in 1X sterile PBS and sterilized through a 0.2 uM sterile membrane filter, and aliquots of stock solution were kept at −20 °C. The 5 mM of working solution of NAC was prepared by diluting stock solution in 1X sterile PBS.

### Nitrite and Nitrate Detection

A Nitrite-Nitrate Test Kit from HACH (Loveland, CO, USA) was used for the detection of nitrite and nitrate concentrations in plasma-treated NAC solution. Plasma treated NAC samples were diluted when required. The results were validated by comparing with standard solutions of nitrite and nitrate following manufacturer’s instructions.

### UV-Visible Spectrum Analysis

UV-visible spectra of solutions were collected. These solutions include the NAC solution that was treated for different time points, the peroxynitrite standard solution, the supernatant (after centrifugation) of bacterial cell suspension after treatment with plasma-treated NAC solution, the peroxynitrite solution and rehydrated (with PBS) liquid of dried remnant of plasma-treated NAC solution. The UV-visible spectra were generated with a UV-visible spectrophotometer (Thermo Scientific, Hudson, NH, USA) between 190 nm and 1100 nm wavelengths with 1 nm interval.

### Fourier Transform Infrared Spectroscopy (FT-IR) Analysis of the Plasma-Treated NAC Solution

Untreated and plasma-treated NAC solutions were evaporated with a rotary evaporator. Infrared spectra of the remaining powders from the evaporation of untreated and 3-minute plasma-treated NAC solutions were obtained with an Olympus BX51 microscopy system (Olympus, Japan) and modified with a Fourier-Transform infrared spectrometer *IlluminatOR* that is equipped with an ATR lens (Smith Detection, USA).

### Nuclear Magnetic Resonance (NMR) of Plasma-Treated NAC Solution

NMR analysis of the NAC solutions was conducted using deuterated water (D_2_O) (Sigma Aldrich, St. Louise, MO, USA). Proton NMR spectra were collected using a Varian INOVA 500 MHz FT-NMR device (Palo Alto, CA, USA). Antimicrobial effect of plasma-treated NAC solutions prepared in D_2_O was also tested in order to assure that its antimicrobial property was same as NAC solution that was prepared in H_2_O.

### Antimicrobial Effect of Acidity and Plasma-Generated Species

In order to determine whether the reduction in pH after plasma treatment is responsible for the disinfection effect, acetic acid (CH_3_COOH), nitric acid (HNO_3_) (Fischer Scientific, Pittsburgh, PA, USA), sulfuric acid (H_2_SO_4_), hydrochloric acid (HCl) and phosphoric acid (H_3_PO_4_) (Sigma Aldrich, St. Louise, MO, USA) solutions were prepared and adjusted pH to ~2.3 (equivalent to 3 min of plasma treatment). The prepared acid solutions were exposed to the same volume of 10^7^ CFU/ml *E. coli* and held for 15 minutes. Then colony-counting assay was performed for the quantification of surviving bacteria as described previously^9^. Also, the antimicrobial effect of plasma-generated species was tested by preparing the determined concentrations of each species in deionized water. For species combination experiments, final concentrations of each species were adjusted accordingly. Hydrogen peroxide solution (30%), nitric acid (1N), glacial acetic acid (Fisher Scientific, Pittsburgh, PA, USA), sodium nitrite and cysteic acid (Sigma Aldrich, St. Louise, MO, USA) were used to prepare desired concentrations of tested substances. Superoxide thermal source (SOTS-1) (Cayman, Ann Arbor, MI, USA) was used to prepare superoxide solution. SOTS-1 is provided as crystalline powder and its stock solution was prepared by dissolving it in dimethyl sulfoxide (DMSO) and stored at −80 °C. The working solution was prepared by dissolving stock solution in PBS. Peroxynitrite (ONOO^−^) (Cayman, Ann Arbor, MI, USA) solution was diluted in deionized water just before exposure to bacteria. Peroxynitrite solution was provided in 0.3 M sodium hydroxide (NaOH). Therefore antimicrobial effects of corresponding concentrations of NaOH were tested in order to make sure that it doesn’t interfere with antimicrobial effect. All species and all combinations of them were exposed to equal volume of 10^7^ CFU/ml *E. coli* and held for 15 minutes. Then colony-counting assay was performed for the quantification of surviving bacteria as described previously^9^.

### Antimicrobial Effect of Components of Plasma-Treated NAC Solution

The NAC solution was prepared by dissolving NAC powder (Sigma) in PBS solution (Sigma). To demonstrate the contribution of two components of plasma-treated NAC solution [NAC molecule (solute) and PBS (solution)], study chemical modifications, and correlate with antimicrobial activity, an experiment was carried out to separate the components of treated NAC solution. In brief, after 3-minute of plasma treatment, the NAC solution was immediately transferred to a glass beaker, the glass beaker was covered with a glass petri dish and heated on a hot plate to 50 °C until all the solvent was evaporated. The evaporated and condensed solvent was collected separately in a microtube. Dried, plasma-treated NAC powder (solute) was reconstituted in three different ways: **1)** with untreated PBS, same volume (equivalent to evaporated liquid) to yield 5 mM final concentration, **2)** with untreated PBS, to half of the volume of evaporated liquid to yield 10 mM final concentration, and **3)** in the same volume of actual evaporated liquid (to give final concentration of 5 mM). Also the condensed liquid part of plasma treated NAC solution was exposed to bacteria in 1:1 (50 μl bacteria: 50 μl solution) and 1:2 (50 μl bacteria: 100 μl solution) ratios. The analyses were carried out for the measurement of pH, antimicrobial activity, and UV-vis spectra. For antimicrobial assays, each liquid was exposed to the equal volume (50 μl:50 μl) of cell suspension containing 10^7^ CFU/ml *E. coli*, held for 15 minutes, and colony counting assay was performed as previously described. Also NAC powder was treated with DBD plasma for 3 minutes and then NAC solution was prepared by dissolving the treated NAC powder in untreated PBS to create a solution of 5 or 10 mM (final concentration). These solutions were exposed to equal volume of 10^7^ CFU/ ml *E. coli,* held for 15 min of holding or contact time, and colony counting assay and pH measurements were performed The final concentrations do not represent how much active species are generated by plasma treatment, but refer to the initial concentration of untreated NAC (5 mM).

### Antimicrobial activity of plasma-treated NAC solution and the effect of diluent

A colony count assay was carried out in order to evaluate persistence of antimicrobial activity of plasma treated NAC solution following dilution with diluent (untreated PBS). In colony count assay experiments plasma-treated NAC solution was mixed with bacteria suspension, held for 15 minutes in order to let the bacteria and NAC solution to come in contact. After 15 minutes of holding time, plasma-treated NAC solution – bacteria suspension mixture was further serially diluted to obtain countable number of bacteria. The possibility of persisted antimicrobial activity of plasma-treated NAC solution following dilution was considered. Therefore, an experiment in which antimicrobial activity of first plasma-treated and then diluted NAC solution was carried out; and the findings are shown in Figure S1.

### Data Analysis

All experiments had built-in negative and positive controls as stated. The initial concentrations (1 × 10^7^ CFU/ml) of bacteria (untreated samples or 0 time treatment samples) were taken as 100% surviving cells to calculate relative percent inactivation (unless specifically stated). All of the experiments were carried out thrice in triplicate. Wherever needed, Prism software v4.03 for Windows (Graphpad, San Diego, CA) was used for analysis. A *P*-value was derived using pair comparisons between two bacterial groups with the Student *t* test and one-way analysis of variance for multiple comparisons. The *P*-value of <0.05 was considered statistically significant.

## Results

### Nitrite and Nitrate Detection

**Figure 1:**
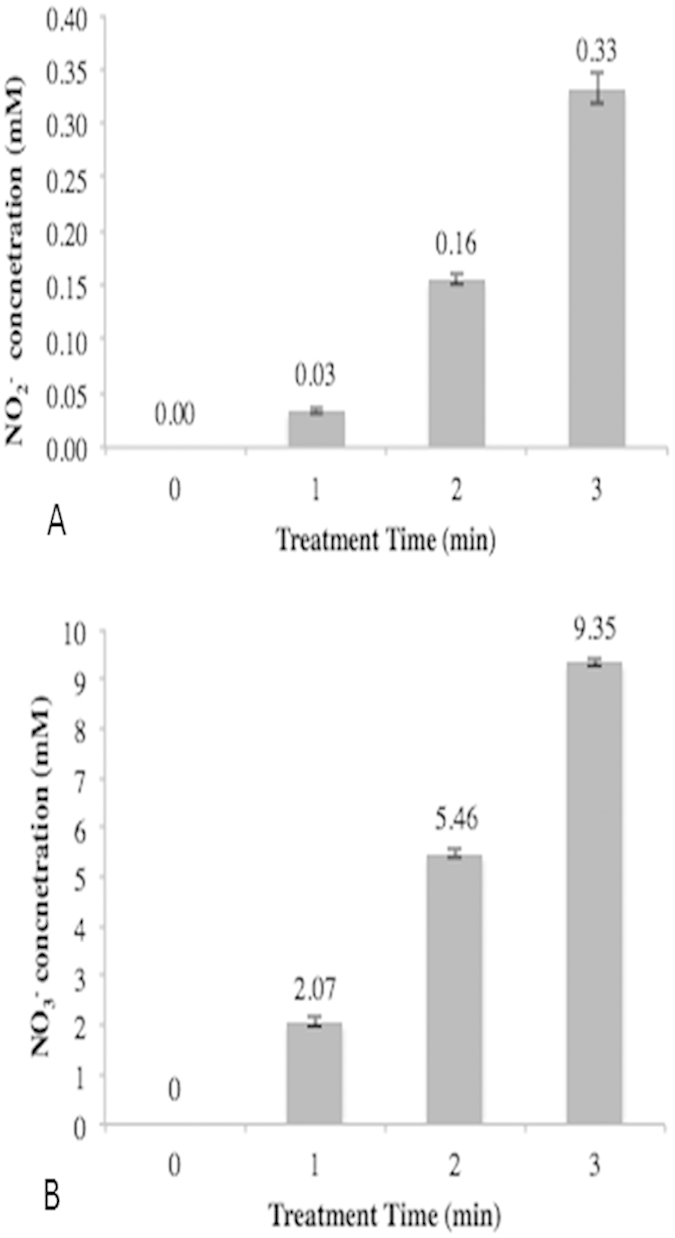
Generation of Nitrite and Nitrate in NAC Solution during Plasma Treatment. Nitrite **(A)** and nitrate concentration **(B)** in plasma-treated NAC solution increases in a plasma treatment time-dependent manner. The data shows relative concentrations to 0 min plasma treatment time (untreated NAC solution). In 3 minute of plasma treatments, the significantly high concentrations of nitrite (0.33 mM) and nitrate (9.35 mM) were detected in plasma treated NAC. (Bar, SD; *p < 0.05; n = 3).

Both nitrite and nitrate concentrations in plasma treated NAC solution increased with plasma treatment time. The nitrite concentrations in plasma treated NAC solution were determined as 0.03 mM, 0.16 mM, and 0.33 mM after 1-minute, 2-minute and 3-minute of plasma treatment, respectively (Fig. 1A). Nitrate concentrations in plasma treated NAC solution were measured as 1 order magnitude more than nitrite, and 2.07 mM, 5.46 mM, and 9.35 mM nitrate were generated after 1, 2 and 3 minutes of plasma treatment, respectively (Fig. 1B).

### UV-Visible Spectrum Analysis of Plasma Treated NAC Solution

**Figure 2:**
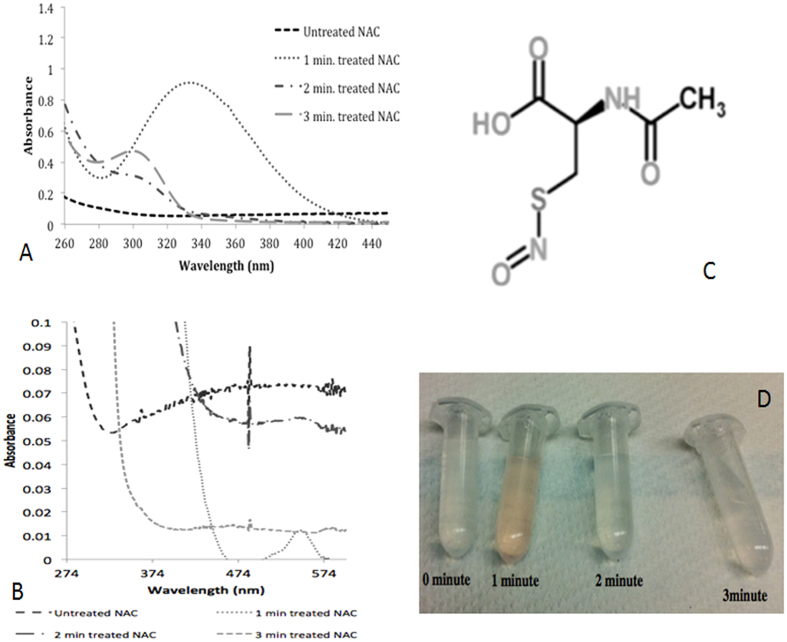
UV-Visible Spectra of Plasma Treated NAC Solution. **(A)** Following 1-minute plasma treatment of NAC solution, a peak at 332 nm (representing formation of S-nitroso N-acetyl cysteine (S-NAC); a type of S-nitrosothiol molecule) was observed. Following 2-minute of plasma treatment, the peak at 332 nm was shifted to 302 nm and the intensity of the peak at 302 nm increased in the plasma treatment time dependent manner. **(B)** Specific secondary peak for S-nitroso N-acetyl cysteine (S-NAC) in 1-minute plasma treated NAC solution. **(C)** S-nitroso N-acetyl cysteine (S-NAC) molecule, H atom is abstracted from thiol group and NO group is bonded after 1-minute of plasma treatment. **(D)** A change in color was observed in 1-minute plasma treated NAC solution, which is characteristic for s-nitrosothiols. Our observation on plasma treated NAC solution along with UV-vis results suggests the formation of S-NAC in 1-minute plasma treated NAC solution.

UV-visible spectra of plasma treated NAC solutions were obtained after 1, 2, and 3 minute of plasma treatments. In the spectrum of 1-minute plasma treated NAC solution, a specific peak appears at 332 nm, which belongs to S-nitroso-N-acetyl cysteine (SNAC) that is a type of S-nitrosothiol. This peak disappears in the spectra of 2 and 3-minute plasma treated NAC solutions. In the spectrum of 2-minute plasma treated NAC solution a new peak starts to appear at 302 nm and the absorbance value of this peak increases after 3 minutes of plasma treatment (Fig. 2A). The specific peak (B), molecular structure (C) and color (D) of S-nitroso-N-acetyl-Cysteine developed during plasma treatment is shown here (Fig. 2).

### FT-IR Analysis of Plasma Treated NAC

**Figure 3:**
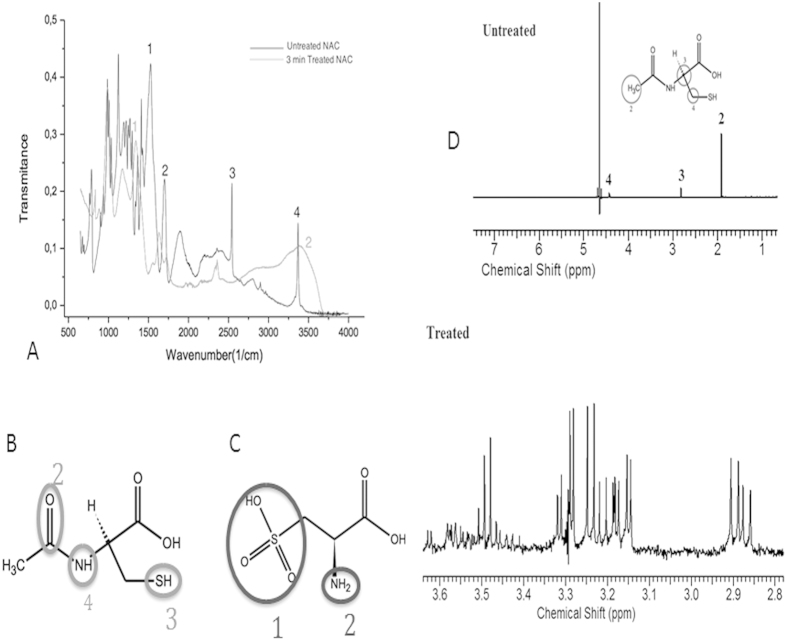
FT-IR and NMR analysis of plasma treated NAC. (**A)** A graphical and schematic diagram showing specific peaks representing NAC molecule in untreated NAC solution, and cysteic acid molecule as a product of NAC in consequence of 3 minute plasma treatment of NAC solution. **(B)** Peaks of untreated NAC molecule at 3375 cm^−1^, 2547 cm^−1^, 1718 cm^−1^, and 1535 cm^−1^ (from Fig. 3A) correspond to the stretching motion of N–H in CONH group (shown with number 4 in spectra and on molecule), S–H (number 3), C = O (number 2) and CONH group, respectively. **(C)** New peaks after plasma treatment appear at 3600–3000 cm^−1^ (Fig. 3A; number 2), and 1344 cm^−1^ (Fig. 3A; number 1), which correspond to the stretching motion of −NH_2_ and −SO_3_H, respectively, which suggest the formation of cysteic acid. **(D)** NMR spectrum of untreated and 3-minute plasma treated NAC solution which shows spin coupling patterns of untreated N-acetyl cysteine (numbered on spectra peaks and molecule), which is being chemically converted to cysteic acid as a result of plasma treatment. Arrows indicate increase in intensity of multiplets found in 3.54–3.61 and 3.24–3.42 ppm. Also, decrease in intensity of the multiplet located at 2.95–3.01 ppm was observed. Proton shifts suggest that about 90% of N-acetyl cysteine is converted to cysteic acid via cleavage of the thiol group.

The graphical presentation of FT-IR data on untreated and 3 min plasma treated NAC is shown (Fig. 3A). The IR peak interpretation of untreated NAC molecule at 3375 cm^−1^, 2547 cm^−1^, 1718 cm^−1^, and 1535 cm^−1^ correspond to the stretching motion of N–H in CONH group, S–H, C = O, and CONH group, respectively are shown (Fig. 3B). The absorptions at these peaks disappear after plasma treatment of NAC solution. New peaks after plasma treatment appear at 3600–3000 cm^−1^ (broad), and 1344 cm^−1^ (narrow), which corresponds to the stretching motion of −NH_2_ and −SO_3_H, respectively are shown in Fig. 3(C). FT-IR spectra of untreated and 3 minutes of plasma treated NAC solution suggest that the SH group was converted to −SO_3_H. Moreover the cleavage of N-acetyl group leads to formation of acetic acid (CH_3_COOH) and –NH_2_ formation due to the cleavage of the of the CONH group. These results suggest that NAC molecule was converted to acetic acid (CH_3_COOH) and a −SO_3_H-containing compound, most likely cysteic acid (C_3_H_7_NO_5_S), due to oxidation of thiol group as a result of plasma treatment. Peaks corresponding to acetic acid cannot be seen on the spectrum because of the evaporation of acetic acid during the evaporation of water before FT-IR analysis.

### Nuclear Magnetic Resonance (NMR) Analysis of Plasma Treated NAC Solution

The NMR spectra of untreated and 3 min plasma treated NAC solution are shown (Fig. 3D). The NMR spectra of the plasma-treated N-acetyl cysteine solution showed that NAC was first oxidized to cysteic acid by cleavage of the N-acetyl group from the NAC molecule and oxidation of the thiol. Increasing duration of plasma treatment of N-acetylcysteine resulted in increase in intensity of multiplets found in 3.54–3.61 and 3.24–3.42 ppm, as well as decrease in intensity of the multiplet located at 2.95–3.01 ppm. The proton shifts suggest that majority of thiol groups of NAC may have been cleaved.

### Antimicrobial Effect of Acidic pH and Plasma-Generated Species

**Figure 4:**
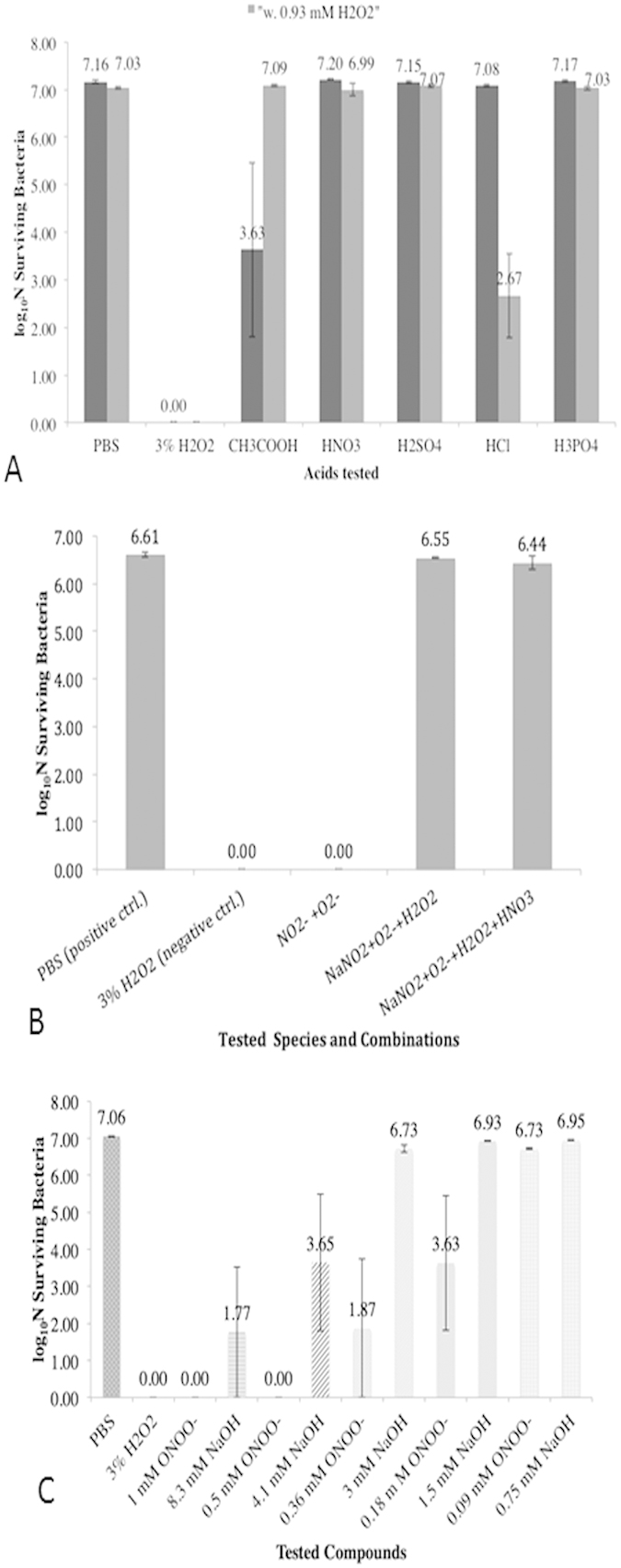
Colony assays showing surviving bacterial cells in response to acids, peroxynitrite and combinations of ROS and RNS, simulating 3-minute plasma treated NAC solution. (**A**) Different acids having equivalent pH (set to 2.3) do not show significant antimicrobial effect, except acetic acid. However, the effect of acetic acid diminishes when hydrogen peroxide is added. With addition of hydrogen peroxide to acids also do not show a significant antimicrobial effect, except hydrochloric acid (which is due to formation of hypochlorous acid). (**B**) Combination of superoxide with nitrite resulted in 7 log reduction of *E. coli*. However, this effect diminishes when hydrogen peroxide and nitrate are added. These results suggest RNS could be the most dominant source for antimicrobial effect in present plasma-treated NAC solution. (**C**) The findings of concentration-dependent antimicrobial effect of peroxynitrite are shown. Since peroxynitrite solution was provided in sodium hydroxide, antimicrobial effect of corresponding sodium peroxide concentration for each tested concentration of peroxynitrite was also included to make sure that presence of sodium hydroxide does not interfere with antimicrobial effect. Peroxynitrite interval (0.18 mM–0.36 mM) showed very high inactivation of *E. coli* suggesting that peroxynitrite might be a major source for antimicrobial effect of plasma treated NAC solution. The conditions of PBS and 3% H_2_O_2_ are shown as positive and negative controls for growth, respectively; and Fig. 4(B) test conditions contain 0.93 mM H_2_O_2_ (the amount detected in 3 min plasma-treated NAC solution).

Figure 4 shows the findings on *E. coli* responses against various conditions that simulate the chemical species/products generated after 3 min plasma treatment. The data in Fig. 4(A) demonstrates that various acids at pH ~2.3 (except acetic acid) alone do not have significant inactivation on 10^7^ CFU/ml of *E. coli.* Acetic acid exhibited ~3.5 log reduction. Acetic acid is a weaker acid compared to other acids that are used in this experiment. Therefore, higher amount of acetic acid needed to obtain pH 2.3 solution of it led to higher concentration of acetate ion (CH_3_CO_2_^−^). Except hydrochloric acid (HCl), when all tested acids were combined with 0.93 mM H_2_O_2_ (the detected concentration of H_2_O_2_ in NAC solution after 3-minute of plasma treatment), did not show significant improvement in antimicrobial effect. The increased antimicrobial effect of HCl when it is combined with H_2_O_2_ may be attributed to the formation of hypochlorous acid, the halogenating agent^34^. In addition, acetic acid loses its antimicrobial activity when it is combined with H_2_O_2_. Similarly, the combinations of nitrate or nitrite with hydrogen peroxide were also tested for their bacterial inactivation property, and the findings suggested that a combination of nitrite and superoxide has excellent bactericidal effect (Fig. 4B).

Table 1(A) summarizes the concentrations of plasma generated products via the interaction of the NAC molecule with plasma discharge in the present study. The concentrations of each given species were tested for their antimicrobial activity in order to reveal an effective contribution of species generated in NAC solution during plasma treatment. Thus, the antimicrobial effects of detected concentrations of nitrite (0.33 mM), nitrate (9.35 mM), hydrogen peroxide (0.93 mM), acetic acid (~5 mM), and cysteic acid (~5 mM) in 3-minute plasma treated NAC solution were tested on 10^7^ CFU/ ml *E. coli* by itself, and in combination with each other. The pH of all tested mixtures was set to ~2.3–2.5. Table 1(B) shows all tested species and their combinations.

None of the tested species or their combinations was not able to show a significant antimicrobial effect and most showed <1 log reduction in CFU. In further experiments, the determined concentrations of H_2_O_2_, NO_3_^−^, and NO_2_^−^ were combined with arbitrarily selected concentrations (0.1, 0.5 and 1 mM) of superoxide that was generated by SOTS-1 (superoxide thermal source) compound. Since the SOTS-1 is dissolved in DMSO, antimicrobial effects of corresponding concentrations of DMSO were also tested in order to make sure that DMSO doesn’t interfere with results. Superoxide in given concentrations and in combination with detected concentration of H_2_O_2_ did not show any significant antimicrobial effect (<1 log reduction in CFU). Similarly, 0.1 mM or 0.5 mM of superoxide with nitrate (9.35 mM) combinations have shown less than 1-log reduction in CFU. Combination of 1 mM superoxide with 9.35 mM nitrate inactivated close to 1 log bacteria. Finally, when the given concentrations of superoxide were combined with 0.33 mM nitrite the results revealed that only combination of 1 mM superoxide with 0.33 mM nitrite was able to achieve more than 6.5-log reduction of CFU. This effect was disappeared when hydrogen peroxide and nitrate were added to superoxide-nitrite mixture (Fig. 4B). These results led us to believe that RNS might be primary reason for the antimicrobial effect of plasma treated NAC solution. Therefore, we tested the antimicrobial effect of determined concentration of peroxynitrite (ONOO^−^) on 10^7^ CFU/ml of *E. coli.* Since the peroxynitrite solution was provided in NaOH solution, the antimicrobial effect of corresponding concentrations of NaOH alone were also tested to ensure that NaOH does not contribute or interfere with antimicrobial effect. As demonstrated in Fig. 4(C), 0.36 mM and 0.18 mM of peroxynitrite showed more than 5.5 and 3.5 log reduction, respectively.

### Antimicrobial Effect of Separated Components of Plasma-Treated NAC Solution

**Figure 5:**
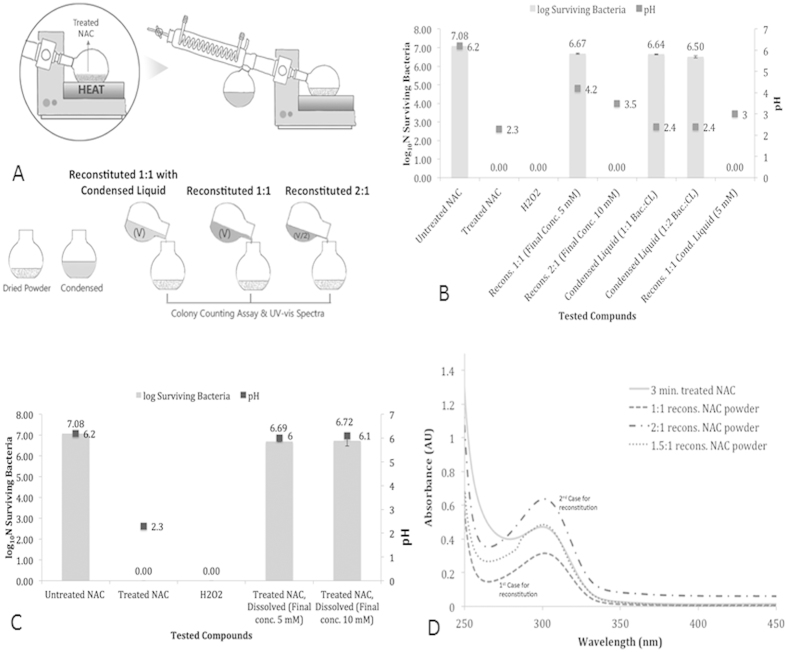
Separation schema and features of plasma-treated NAC solution components. **(A)** Separation schema of plasma-treated NAC solution to liquid portion and solute portion. Plasma-treated NAC solution was heated; evaporated liquid portion was condensed and collected separately. Remaining solid (powder) portion was reconstituted using untreated PBS solution in different ratios or in condensed liquid portion. **(B)** Colony assays were performed using reconstituted samples following separation of 3-minue plasma-treated NAC solution. When separated dried NAC portion was reconstituted in 1:1 ratio (to achieve final [5 mM] NAC) using untreated PBS, no significant microbial inactivation was observed; but solution prepared using condensed liquid to untreated PBS in 2:1 ratio (means remaining powder concentration was doubled) resulted as 7 log reduction (complete inactivation). The pH values of each condition are also shown. **(C)** Colony assays showing fresh NAC powder, treated with plasma-discharge for 3 minutes by itself, then dissolved in untreated PBS to obtain 5 mM and 10 mM final concentration of NAC, the solution did not show significant antimicrobial effect. **(D)** UV-visible spectrum of 3-minute plasma-treated NAC solution, 1:1 and 2:1 ratio reconstituted samples and 1.5:1 ratio reconstituted sample to obtain same intensity of the peak at 302 nm that was obtained from 3-minute plasma treated NAC solution using Beer-Lambert Law.). Following reconstitution (1:1 ratio of plasma-treated NAC solution; after heating and evaporation of liquid part), intensity of the peak at 302 nm relatively decreased (explained in manuscript as 1^st^ condition). When remaining powder was reconstituted in 2:1 ratio (to double the concentration of remaining powder/ dried portion), the intensity of peak was doubled (explained in manuscript as 2^nd^ condition), suggesting the products of NAC molecule probably stabilized peroxynitrite. When remaining powder (dried portion) was reconstituted in 1.5:1 ratio the same intensity of the peak at 302 nm (obtained from 3-minute plasma treated NAC solution) was observed; and no hydrogen peroxide, no nitrite and 0.8 mM nitrate (<10% what was detected in 3-minute plasma-treated NAC solution) was detected, and 7 log reduction of cells was achieved. These findings suggest that peroxynitrite is a major source for microbial inactivation.

As explained in the materials and methods section, the schema of separation of components of plasma-treated NAC solution is depicted in Fig. 5(A). The different methods were used for reconstitution of plasma treated NAC solutions. In the first case no antimicrobial effect was observed. In the second and third cases 7-log reduction was observed. When condensed liquid portion of plasma treated NAC solution alone was used, no inactivation was observed. The pH of reconstituted solutions was found to be slightly increased and measured as 4.2, 3.5, and 3 for the 1^st^, 2^nd^, and 3^rd^ cases respectively. Also, the condensed liquid retained its acidity, where the pH was measured as 3. Figure 5(B) summarizes the inactivation and pH measurement results of the reconstitution experiments. In case of DBD plasma treatment of NAC powder by itself and preparation of NAC solution with plasma-treated NAC powder (dried crust), no inactivation effect was achieved, with both 5 mM and 10 mM solutions. The pH of prepared solutions were measured as ~6 (Fig. 5C). The UV-visible spectra obtained from the reconstitution experiments revealed that the intensity of the peak at 302 nm is directly related to the concentration of peroxynitrite. In other words, after heating, the peak at 302 nm decreases and is therefore attributable to liquid that was removed by the evaporation process. When the remaining powder is reconstituted with PBS in a 1:1 ratio (same as the evaporated liquid), the absorbance of the peak at 302 nm is measured as 0.31, and when the powder is reconstituted with PBS in a 2:1 ratio (half volume of the evaporated liquid), the peak at 302 nm is measured as 0.62. With a basic dilution calculation we calculated that if the dried powder is reconstituted with PBS in a 1.5:1 ratio (0.75 times of evaporated volume), the peak intensity should be same as the 3-minute plasma treated NAC solution. Therefore, the dried NAC powder was reconstituted with PBS in a 1.5:1 ratio, its antimicrobial effect was tested using colony counting assay, and nitrite, nitrate and hydrogen peroxide concentrations were measured using HACH nitrite/nitrate detection kit and hydrogen peroxide assay kit, and the UV-visible spectrum was obtained. Figure 5(D) demonstrates that all tested solutions show intensity of the peak at 302 nm, and at this peak, a similar absorbance (0.47 AU) is observed by 3-minute plasma treated NAC solution and the dried powder reconstituted solution of 1.5:1 ratio. In addition, the dried powder that was reconstituted in 1.5:1 ratio had achieved 7-log reduction, and nitrate, nitrite and hydrogen peroxide concentrations were measured as 0.8 mM, 0 mM, and 0 mM respectively. Through these results we speculate that the peak at 302 nm represents peroxynitrite and products of the NAC or unreacted NAC molecule somehow act similar to a spin trap and play a role in the stabilization of plasma generated reactive species. However the stabilization process is unclear and further studies are required in order to understand the mechanism of stabilization of plasma-generated species.

Since we postulate the presence of peroxynitrite in plasma treated NAC solution from our findings, the concentration of peroxynitrite in plasma treated NAC solution was calculated by using the Beer-Lambert Law. The extinction coefficient of peroxynitrite (ε_ONOO_) is given as 1670 M^−1^.cm^−1^ 1,27,36,37,38,39 and concentration of peroxynitrite in 3-minute plasma treated NAC solution was calculated as 0.28 mM. UV-visible spectra of reconstituted solutions showed a specific peak at 302 nm similar to plasma treated liquid. Peaks at 302 nm represent ONOO^−^, and concentrations of ONOO^−^ calculated as 0.18 and 0.36 mM for the above-mentioned 1^st^ and 2^nd^ reconstitution cases respectively (Fig. 5D).

## Discussion

According to our findings, the antimicrobial effect of plasma treated NAC solution seems to be from the chemical modifications in NAC solution during plasma treatment. The previous literature which have reported a moderate reduction in pH of PBS or water always had a plasma discharge gap of more than 2 mm through 45 mm, and the amount of liquid more than 1.5 ml through 10 ml^10^,^16^,^17^,^20^. This drastically changes the chemical properties and pH equation of these solutions. Our findings show that acidic pH has no direct significant contribution to bacterial inactivation. Similar observations are also reported by other groups^10^,^20^,^21^. However, acidic pH seems to be a crucial for the antimicrobial effect of the solution, and usually can be referred to antimicrobial effect of acidified nitrites^30^,^31^,^32^.

During analysis, we did not see significant antimicrobial activity at the detected concentrations of plasma-generated species alone. In partly, this might be due to lack of sufficient concentration of individual plasma-generated species. Plasma treated liquids are complex mixtures of ROS and RNS and carry various charged and uncharged molecules. Further detailed chemical analyses and chemical kinetic experiments are required to better understand the exact roles of plasma-generated species on microbial inactivation. Oehmigen *et al*. have reported that the mixture of 0.074 mM H_2_O_2_ and 0.032 mM NO_2_^−^ at pH 3 is able to inactivate 3.5 logs *E. coli*^15^. However this effect is mostly related to exposure conditions of bacteria to hydrogen peroxide-nitrite mixture. In their study, bacteria were exposed 50 times more volume of mixture (50 μl bacteria: 2.45 ml mixture) as opposed to our 1:1 (50 μl bacteria: 50 μl mixture or plasma treated NAC) ratio and held for 60 minutes as opposed to our 15 minutes of holding time. Therefore our findings on the nitrite-superoxide mixture, which demonstrates 7 log reductions under the similar exposure conditions with plasma treated NAC, seems to be more significant, and relevant to our plasma treatment method. In addition, the inactivation rate of peroxynitrite in the detected range supports our hypothesis of prominent contribution of RNS in *E. coli* inactivation. However precise quantification of superoxide is required for better understanding of the interactions between oxygen and nitrogen species.

An interesting chemical modification was observed in 1-minute plasma treated NAC solution. In the UV-visible spectrum of 1-minute plasma treated NAC solution, we saw a peak at 332 nm, specific for S-nitrosothiols (in our case S-nitroso-N-acetyl cysteine). Also, S-nitrosothiols have another specific peak at 545 nm with 16 M^−1^ cm^−1^ molar extinction coefficient, which is more suitable for concentration calculation due to lack of possible interference by other nitrogen species^40^. According to the absorbance of the peak at 545 nm and the molar extinction coefficient, the concentration of S-nitroso-N-acetyl-cysteine is calculated as 0.75 mM by using Beer-Lambert equation (Fig. 2B). S-nitrosothiols, with the general structure of RSNO (R is an organic group) are the S-nitrosylated products of thiols and are known NO (nitric oxide) donors and carriers, with a characteristic pink color^2^,^41^,^42^. Our findings and observations on 1 minute plasma treated NAC solution regarding the formation of S-nitroso-N-acetyl cysteine (Fig. 2C) is consistent, and supported by previous literature. We observed a pinkish color development in 1 minute plasma treated NAC solution (Fig. 2D). Formation of S-nitrosothiols (RSNO) involves the reaction of a thiol (RSH) with NO and NO derivatives such as NO_2_, NO_2_^−^, and N_2_O_3_. By itself NO reaction with a thiol leads to disulfide formation rather than RSNO. However, in the presence of oxygen or other oxygen species such as hydrogen peroxide, NO oxidation leads to formation of S-nitrosothiols^43^. Oxidation of thiols with plasma generated ROS involves the production of sulfonyl radical that later react with NO to form S-nitrosothiol as shown in the equations below^44^.




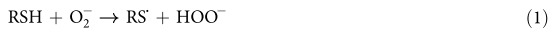







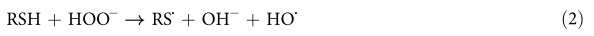







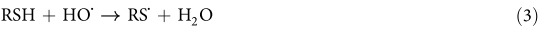




Thus, plasma generated ROS and RNS cause the modification of NAC to S-NAC by the end of 1-minute plasma treatment. The interaction of O_2_^−^ and NO leads to peroxynitrite (ONOO^−^). S-nitroso-N-acetyl cysteine acts as NO donor via decomposition for peroxynitrite formation in plasma treated NAC solution. Not only plasma generated ROS and RNS but also UV light that is generated during plasma treatment, and acidic pH as a result of plasma treatment of liquids, have influence on the production and the decomposition of SNAC. UV light and the presence of thiols (unreacted NAC) in acidic environment induce decomposition of S-nitrosothiols (in our case S-NAC)^42^. Nitric oxide^5^ is released via decomposition of S-NAC and reacts with plasma-generated superoxide (O_2_^−^) for peroxynitrite formation. Another mechanism for the S-nitrosothiol mediated peroxynitrite formation which is relevant to plasma treated liquids, involves the reaction of hydrogen peroxide with S-NAC. This mechanism involves the dissociation of hydrogen peroxide and the reaction of hydroperoxyl (HO_2_^−^) with S-NAC as shown in the following equations^45^.




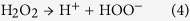







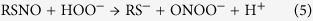




In addition to plasma-generated superoxide, by itself the S-nitrosothiol formation mechanism can lead to superoxide production via reduction of O_2_ by RSNO-H, a radical intermediate, as shown in following equations^46^.




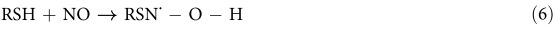







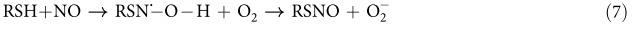




As stated above, in the plasma treated NAC sample that was dried and reconstituted in 1.5:1 ratio, hydrogen peroxide and nitrite was totally diminished and nitrate concentration was measured as 0.8 mM, which in contrast, is less than 10% of the nitrate concentration found in 3-minute plasma treated NAC solution. This sample was capable of 7 log reduction, supporting our speculation on the presence of peroxynitrite, and supports its relation with the peak at 302 nm.

Our FT-IR and NMR data suggests that about ~90% NAC is converted to cysteic acid. This mechanism involves the oxidation of NAC as given in equations 1, 2, 3. Oxidation of thiols results in formation of disulfides that later are likely to be oxidized to sulfonic acid (in our case cysteic acid)^44^,^47^. In such reaction, where NAC is converted to cysteic acid, acetic acid is also formed due to cleavage of acetyl group. The overall reaction is shown in the equation below:




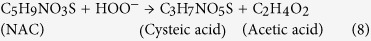




It can be observed from equation 8 that in order to fulfill the atomic balance in the reaction, NAC should react with HOO^−^ which is present in the plasma treated NAC solution, probably due to the dissociation of H_2_O_2_ as shown in equation 4 or protonation of superoxide. This possible reaction mechanism explains the decreased hydrogen peroxide concentration in 3 minute plasma treated of NAC solution compared to 2-minute plasma treated NAC solution. The formation of cysteic acid and acetic acid contributes to the rapid and drastic pH drop in NAC solution following plasma treatment.

## Conclusion

The findings suggest that plasma treatment turns NAC solution into an acidic mixture of ROS and RNS where both contribute to bacterial inactivation. NAC seems to be in the center of all reactions and interactions between ROS and RNS. NAC by itself serves as a source of RNS by releasing NO, and also the source of ROS through the intermediates upon its reaction with other ROS. Although the antimicrobial effect can be attributed to both ROS and RNS, based on our results we speculate that ROS play an important role in the modification of NAC molecule, while RNS seems to contribute to the antimicrobial effect more dominantly. This is the first report to our knowledge of the generation of peroxynitrite during nonthermal plasma treated NAC solution which is capable of inactivating7 log of CFU of *E. coli*. Further studies on the mechanism of action ROS and RNS on microbial inactivation and response of bacteria to those species are underway.

## Additional Information

**How to cite this article**: Ercan, U. K. *et al*. Chemical Changes in Nonthermal Plasma-Treated N-Acetylcysteine (NAC) Solution and Their Contribution in Bacterial Inactivation. *Sci. Rep.* **6**, 20365; doi: 10.1038/srep20365 (2016).

## Supplementary Material

Supplementary Information

## Figures and Tables

**Table 1:** **C**hemistry of plasma treated NAC solution.

**[A]**					
**Compound**	**Concentration [mM]**				
Hydrogen peroxide (H_2_O_2_)	0.93				
Nitrite (NO_2_^−^)	0.33				
Nitrate (NO_3_^−^)	9.35				
Acetic acid (CH_3_COOH) (A.A.)	~5				
Cysteic acid (C_3_H_7_NO_5_S) (C.A.)	~5				

**[B]**					
**Single species**	**Combination of 2 species**		**Combination of 3 species**		**Combination of 4 species**
H_2_O_2_	H_2_O_2_ + NO_2_^−^	NO_2_^−^ + A.A.	H_2_O_2_ + NO_2_^−^ + NO_3_^−^	NO_2_^−^ + A.A + C.A.	H_2_O_2_ + NO_2_^−^ + NO_3_^−^ + A.A.
NO_2_^−^	H_2_O_2_ + NO_3_^−^	NO_3_^−^ + A.A.	H_2_O_2_ + NO_2_^−^ + A.A.	NO_3_^−^ + A.A + C.A.	H_2_O_2_ + NO_2_^−^ + NO_3_^−^ + C.A.
NO_3_^−^	H_2_O_2_ + A.A.	NO_2_^−^ + C.A.	H_2_O_2_ + NO_2_^−^ + C.A.	H_2_O_2_ + A.A + C.A.	NO_2_^−^ + NO_3_^−^ + A.A. + C.A.
A.A.	H_2_O_2_ + C.A.	NO_3_^−^ + C.A.	NO_2_^−^ + NO_3_^−^ + A.A.	H_2_O_2_ + NO_3_^−^ + A.A.	A.A. + C.A. + H_2_O_2_ + NO_2_^−^
C.A.	NO_2_^−^ + NO_3_^−^	A.A. + C.A.	NO_2_^−^ + NO_3_^−^ + C.A.	H_2_O_2_ + NO_3_^−^ + C.A.	A.A. + C.A. + H_2_O_2_ + NO_3_^−^
Combination of 5 species: H_2_O_2_ + NO_2_^−^ + NO_3_^−^ + A.A. + C.A.					

**[A]** Species detected in 3-minute plasma treated NAC solution and their concentrations. [**B]** To simulate 3-minute plasma treated NAC solution, antimicrobial activities of detected species were determined either alone or in combination with each other.

The tested combinations of detected species are shown here.

## References

[b1] Holthoff J. H. . Resveratrol, a dietary polyphenolic phytoalexin, is a functional scavenger of peroxynitrite. Biocheml Pharmacol 80, 1260–1265 (2010). 10.1016/j.bcp.2010.06.027 PMC2934873 20599800

[b2] Tsikas D. . S-transnitrosylation of albumin in human plasma and blood *in vitro* and *in vivo* in the rat. BBA-Protein Struct M 1546, 422–434 (2001). 10.1016/s0167-4838(01)00166-2 11295447

[b3] Joshi S. G. . Nonthermal dielectric-barrier discharge plasma-induced inactivation involves oxidative DNA damage and membrane lipid peroxidation in *Escherichia coli*. Antimicrob Agents Chemother 55, 1053–1062 (2011). 21199923 10.1128/AAC.01002-10 PMC3067084

[b4] Cooper M., Fridman G., Fridman A. & Joshi S. G. Biological responses of *Bacillus stratosphericus* to floating electrode-dielectric barrier discharge plasma treatment. J Appl Microbiol 109, 2039–2048 (2010). 20825520 10.1111/j.1365-2672.2010.04834.x

[b5] Gallagher M. J. . Rapid inactivation of airborne bacteria using atmospheric pressure dielectric barrier grating discharge. IEEE T Plasma Sci 35, 1501–1510 (2007).

[b6] Elmoualij B. . Decontamination of Prions by the Flowing Afterglow of a Reduced-pressure N_2_-O_2_ Cold-plasma. Plasma Process Polym 9, 612–618 (2012).

[b7] Laroussi M. & Leipold F. Evaluation of the roles of reactive species, heat, and UV radiation in the inactivation of bacterial cells by air plasmas at atmospheric pressure. Int J Mass Spectrom 233, 81–86 (2004).

[b8] Liang Y. D. . Rapid Inactivation of Biological Species in the Air using Atmospheric Pressure Nonthermal Plasma. Environ Sci Technol 46, 3360–3368 (2012). 22385302 10.1021/es203770q

[b9] Ercan U. K. . Nonequilibrium Plasma-Activated Antimicrobial Solutions are Broad-Spectrum and Retain their Efficacies for Extended Period of Time. Plasma Process Polym 10, 544–555 (2013).

[b10] Naitali M., Kamgang-Youbi G., Herry J. M., Bellon-Fontaine M. N. & Brisset J. L. Combined Effects of Long- Living Chemical Species during Microbial Inactivation Using Atmospheric Plasma- Treated Water. Appl Environ Microbiol 76, 7662–7664 (2010). 20889799 10.1128/AEM.01615-10 PMC2976197

[b11] Burlica R., Kirkpatrick M. J. & Locke B. R. Formation of reactive species in gliding arc discharges with liquid water. J Electrostat 64, 35–43 (2006).

[b12] Chen C. W., Lee H. M. & Chang M. B. Influence of pH on inactivation of aquatic microorganism with a gas-liquid pulsed electrical discharge. J Electrostat 67, 703–708 (2009).

[b13] Traylor M. J. . Long-term antibacterial efficacy of air plasma-activated water. J Phys D Appl Phys 44 (2011).

[b14] Ikawa S., Kitano K. & Hamaguchi S. Effects of pH on Bacterial Inactivation in Aqueous Solutions due to Low-Temperature Atmospheric Pressure Plasma Application. Plasma Process Polym 7, 33–42 (2010).

[b15] Oehmigen K. . Estimation of Possible Mechanisms of *Escherichia coli* Inactivation by Plasma Treated Sodium Chloride Solution. Plasma Process Polym 8, 904–913 (2011).

[b16] Oehmigen K. . The Role of Acidification for Antimicrobial Activity of Atmospheric Pressure Plasma in Liquids. Plasma Process Polym 7, 250–257 (2010).

[b17] Kamgang-Youbi G. . Microbial inactivation using plasma-activated water obtained by gliding electric discharges. Lett Appl Microbiol 49, 292–292 (2009). 10.1111/j.1472-765X.2008.02476.x 19170858

[b18] Julak J., Scholtz V., Kotucova S. & Janouskova O. The persistent microbicidal effect in water exposed to the corona discharge. Phys Medica 28, 230–239 (2012). 10.1016/j.ejmp.2011.08.001 21925912

[b19] Burlica R. & Locke B. R. Pulsed plasma gliding-arc discharges with water spray. IEEE T Ind Appl 44, 482–489 (2008).

[b20] Machala Z. . Formation of ROS and RNS in Water Electro-Sprayed through Transient Spark Discharge in Air and their Bactericidal Effects. Plasma Process Polym 10, 649–659 (2013).

[b21] Von Woedtke T. . Plasma Liquid Interactions: Chemistry and Antimicrobial Effects. In: Machala Z., Hensel K., Akishev Y. editors. “Plasma for Bio-Decontamination, Medicine and Food Security. NATO Science for Peace and Security Series-A: Chemistry and Biology.” Springer, Dordrecht, Netherlands, 2011 (2011).

[b22] Chen C. W., Lee H. M. & Chang M. B. Inactivation of aquatic microorganisms by low-frequency AC discharges. IEEE T Plasma Sci 36, 215–219 (2008).

[b23] Liu F. X. . Inactivation of Bacteria in an Aqueous Environment by a Direct-Current, Cold-Atmospheric-Pressure Air Plasma Microjet. Plasma Process Polym 7, 231–236 (2010).

[b24] Anber M. & Taube H. Interaction of Nitrous Acid with Hydrogen Peroixde and with Water. J Am Chem Soc 76, 6243–6247 (1954).

[b25] Pacher P., Beckman J. S. & Liaudet L. Nitric oxide and peroxynitrite in health and disease. Physiol Rev 87, 315–424 (2007). 17237348 10.1152/physrev.00029.2006 PMC2248324

[b26] Lukes P., Dolezalova E., Sisrova I. & Clupek M. Aqueous-phase chemistry and bactericidal effects from an air discharge plasma in contact with water: evidence for the formation of peroxynitrite through a pseudo-second-order post-discharge reaction of H_2_O_2_ and HNO_2_. Plasma Sources Sci T 23, (2014).

[b27] Marla S. S., Lee J. & Groves J. T. Peroxynitrite rapidly permeates phospholipid membranes. Proc Natl Acad Sci USA 94, 14243–14248 (1997). 9405597 10.1073/pnas.94.26.14243 PMC24925

[b28] Voetsch B., Jin R. C. & Loscalzo J. Nitric oxide insufficiency and atherothrombosis. Histochem Cell Biol 122, 353–367 (2004). 15338226 10.1007/s00418-004-0675-z

[b29] Hughes M. N. Relationships between nitric oxide, nitroxyl ion, nitrosonium cation and peroxynitrite. BBA-Bioenergetics 1411, 263–272 (1999). 10320662 10.1016/s0005-2728(99)00019-5

[b30] Castellani A. G. & Niven C. F. Jr. Factors Affecting Bacteriostatic Action of Sodium Nitrite. Appl Microbiol 3, 154–159 (1955). 14377383 10.1128/am.3.3.154-159.1955 PMC1057086

[b31] Benjamin N. . Stomach NO Synthesis. Nature 368, 502–502 (1994). 8139683 10.1038/368502a0

[b32] Duncan C. . Protection against oral and gastrointestinal diseases: Importance of dietary nitrate intake, oral nitrate reduction and enterosalivary nitrate circulation. Comp Biochem Phys A 118, 939–948 (1997). 10.1016/s0300-9629(97)00023-6 9505412

[b33] Xia D. S., Liu Y., Zhang C. M., Yang S. H. & Wang S. L. Antimicrobial effect of acidified nitrate and nitrite on six common oral pathogens *in vitro*. Chinese Med J-Peking 119, 1904–1909 (2006). 17134590

[b34] McDonnell G. E. Antisepsis, Disinfection and Sterilization: Types, Action and Resistance, ASM Press, Washington DC 2007 (2007).

[b35] Weller R., Price R. J., Ormerod A. D., Benjamin N. & Leifert C. Antimicrobial effect of acidified nitrite on dermatophyte fungi, Candida and bacterial skin pathogens. J Appl Microbiol 90, 648–652 (2001). 11309079 10.1046/j.1365-2672.2001.01291.x

[b36] Lobysheva I. I., Serezhenkov V. A. & Vanin A. F. Interaction of Peroxynitrite and Hydrogen Peroxide with Dinitrosyl Iron Complex Contaning Thiol Ligands in vitro. Biochemnistry (Moscow) 24, 194–200 (1999). 10187905

[b37] Whiteman M., Szabo C. & Halliwell B. Modulation of peroxynitrite and hypochlorous acid-induced inactivation of α1-antiproteinase by mercaptoethylguanidine. Brit J Pharmacol. 126, 1646–1652 (1999). 10323598 10.1038/sj.bjp.0702465 PMC1565935

[b38] Kuhn D. M., Aretha C. W. & Geddes T. J. Peroxynitrite inactivation of tyrosine hydroxylase: mediation by sulfhydryl oxidation, not tyrosine nitration. The J Neurosci 19, 10289–10294 (1999). 10575026 10.1523/JNEUROSCI.19-23-10289.1999 PMC6782408

[b39] McLean S., Bowman L. A. & Poole R. K. KatG from *Salmonella typhimurium* is a peroxynitritase. FEBS letters 584, 1628–1632 (2010). 20307539 10.1016/j.febslet.2010.03.029

[b40] Dykhuizen R. S. . *Helicobacter pylori* is killed by nitrite under acidic conditions. Gut 42, 334–337 (1998). 9577337 10.1136/gut.42.3.334 PMC1727019

[b41] Wang P. G. . Nitric oxide donors: Chemical activities and biological applications. Chem Rev 102, 1091–1134 (2002). 11942788 10.1021/cr000040l

[b42] Hu T. M. & Chou T. C. The kinetics of thiol-mediated decomposition of S-nitrosothiols. Aaps J 8, E485–E492 (2006). 17025266 10.1208/aapsj080357 PMC2761055

[b43] Kharitonov V. G., Sundquist A. R. & Sharma V. S. Kinetics of Nitrosation of Thiols by Nitric-Oxide in the Presence of Oxygen. J Biol Chem 270, 28158–28164 (1995). 7499306 10.1074/jbc.270.47.28158

[b44] Koval I. V. Reactions of thiols. Russ J Org Chem 43, 319–346 (2007).

[b45] Coupe P. J. & Williams D. L. H. Formation of peroxynitrite from S-nitrosothiols and hydrogen peroxide. J Chem Soc Perk T 2, 1057–1058 (1999).

[b46] Gow A. J., Buerk D. G. & Ischiropoulos H. A novel reaction mechanism for the formation of S-nitrosothiol *in vivo*. J Biol Chem 272, 2841–2845 (1997). 9006926 10.1074/jbc.272.5.2841

[b47] Abedinzadeh Z., Arroub J. & Gardesalbert M. On N-Acetylcysteine 2. Oxidation of N-Acetylcysteine by Hydrogen-Peroxide - Kinetic-Study of the Overall Process. Can J Chem 72, 2102–2107 (1994).

