# Supplementary material for: Chemical Changes in Nonthermal Plasma-Treated N-Acetylcysteine (NAC) Solution and Their Contribution to Bacterial Inactivation
Source: Sci Rep. 2016 Feb 2;6:20365. doi: 10.1038/srep20365 (PMC4735827; doi:10.1038/srep20365)
Supplement: Supplementary Information [file srep20365-s1.pdf]

Manuscript: SREP-15-19127B

Chemical Changes in Nonthermal Plasma-Treated N-Acetylcysteine (NAC) Solution and Their Contribution to Bacterial Inactivation

Utku K. Ercan[1§], Josh Smith[2], Hai-Feng Ji[2], Ari D. Brooks[1†], Suresh G. Joshi[1,3*]

Center for Surgical Infection and Biofilm, [1]Department of Surgery, [2]Department of Chemistry, [3]Department Microbiology and Immunology, Drexel University, Philadelphia, PA 19102 USA

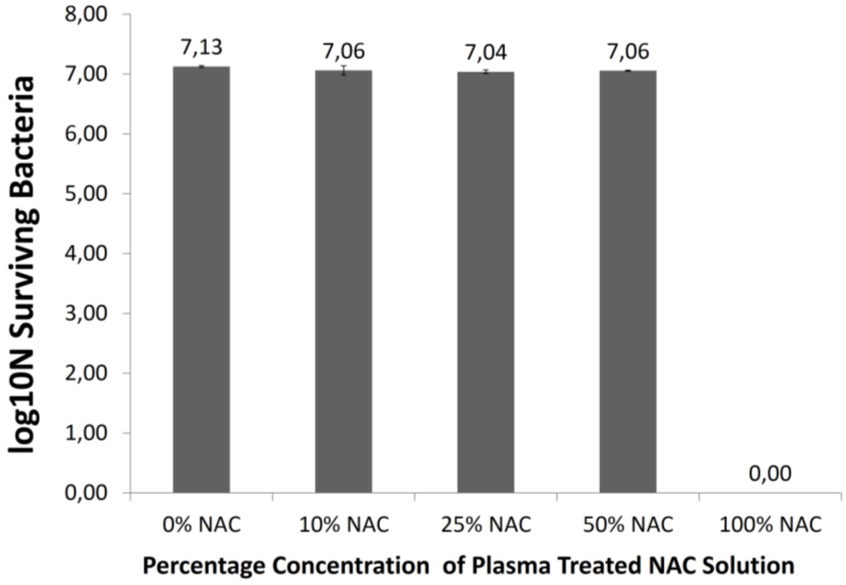

Figure S1. Antimicrobial activity of plasma-treated NAC solution and the effect of diluent. A colony count assay demonstrating the effect of diluent (untreated PBS; PBS) on antimicrobial activity of plasma-treated NAC solution. The possibility of persistence of antimicrobial activity of plasma-treated NAC solution following dilution was considered. Therefore, an experiment in which antimicrobial activity of first plasma-treated and then diluted NAC solution was carried out (please material and methods). Our results presented above demonstrate that the antimicrobial activity of plasma-treated NAC solution disappears when it is diluted even shortly before exposing to bacteria. Thus, the findings also indicate that addition of external scavenger or inhibitor of antimicrobial activity is not required and the antimicrobial activity of plasma-treated NAC solution does not persist after diluting in PBS during plating of bacteria sample.
